# Vaccinations and Functional Feed Supplements as Alternatives to Coccidiostats for the Control of Coccidiosis in Raising Broiler Chickens

**DOI:** 10.3390/ani15172548

**Published:** 2025-08-30

**Authors:** Maciej Rosłoń, Edward Majewski, Monika Gębska, Anna Grontkowska, Michał Motrenko, Artur Żbikowski, Monika Michalczuk, Jakub Urban

**Affiliations:** 1 MBA in Agribusiness Management, Warsaw University of Life Sciences, 02-787 Warsaw, Poland; roslon.maciej@wp.pl; 2 Institute of Economics and Finance, Warsaw University of Life Sciences, 02-787 Warsaw, Poland; edward_majewski@sggw.edu.pl (E.M.); anna_grontkowska@sggw.edu.pl (A.G.); 3 Faculty of Animal Breeding, Bioengineering and Conservation, Warsaw University of Life Sciences, 02-786 Warsaw, Poland; s202889@sggw.edu.pl; 4 Institute of Veterinary Medicine, Warsaw University of Life Sciences, 02-776 Warsaw, Poland; artur_zbikowski@sggw.edu.pl; 5 Institute of Animal Sciences, Warsaw University of Life Sciences, 02-786 Warsaw, Poland; monika_michalczuk@sggw.edu.pl

**Keywords:** coccidiosis, coccidiostats, broiler chickens, vaccinations, functional feed supplements

## Abstract

It is estimated that global meat production will show an upward trend, with the most dynamic growth projected in the poultry sector (it is estimated that poultry meat consumption will be 2.3 times higher by 2050 than in 2010). The expected increase in consumption of poultry meat, mainly from intensively reared broiler chickens, is associated with an increasing prevalence of diseases, particularly those affecting the digestive system. One important parasitic disease is coccidiosis, a gastrointestinal disease caused by widespread protozoa of the genus *Eimeria*. The occurrence of coccidiosis in broiler chicken flocks results in a significant deterioration of production rates. Coccidiostats are most commonly used in the prevention of this disease, which are introduced in rotation into the feed ration. However, long-term use of coccidiostats is associated with the risk of parasite resistance development and the possibility of residues in animal products. Therefore, there is a need to search for safe and effective alternatives to pharmacological coccidiostatic agents. This review aims to analyze the available literature data on the efficacy of vaccines and functional feed supplements, such as plant substances, probiotics, prebiotics, and organic acids, in the prevention of coccidiosis.

## Simple Summary

Global meat production is projected to continue to rise, with the poultry sector expected to expand most rapidly. The growing demand for poultry meat, particularly from intensive broiler chicken systems, is linked to a higher incidence of diseases, especially those affecting the digestive system. Among the most significant threats is coccidiosis, a widespread parasitic disease caused by protozoa of the genus *Eimeria*. Infection in chicken broiler flocks leads to significant deterioration of production performance. To prevent coccidiosis, coccidiostats are routinely incorporated into feed mixtures, often in rotation. However, prolonged use of these agents poses challenges, including the development of parasite resistance and the accumulation of drug residues in poultry tissues. Consequently, there is a growing need for effective and safe alternatives to conventional pharmacological approaches. This review aims to analyze literature data on the effectiveness of vaccines and functional feed supplements, such as plant-derived substances, probiotics, prebiotics, and organic acids, in the prevention and control of coccidiosis.

## 1. Introduction

In recent decades, global meat consumption has risen markedly, and projections indicate that this upward trend will continue. Since the 1970s, global meat production has steadily expanded, increasing by only 20% by the year 2000. Global pork meat production has increased by about 290%; sheep and goat meat by 200%; beef and buffalo meat by 180%; and poultry meat by 700% [1,2]. According to the FAO’s 2006 projections, poultry meat consumption will be 2.3 times higher by 2050 than in 2010. Globally, daily calorie supply will increase from 2712 to 3226 between 2010 and 2050 [3]. However, publicly available forecasts suggest that annual meat production will need to increase by more than 200 million tonnes to reach 470 million tonnes in 2050 [1,2].

Poultry meat production mainly uses highly selected broiler chicken hybrids, which are primarily intended for rearing in intensive production systems. Over the last 30 years, the slaughter weight of broiler chickens has doubled, while the rearing period has been reduced by almost half. Unfortunately, genetic progress in broiler chicken selection has contributed, among other things, to the thinning of the intestinal mucosa [4,5], which in turn increases the risk of coccidiosis in broiler chicken flocks. One of the main roles of mucosal surfaces is to protect the body against a huge number and variety of harmful antigens, and the primary role of GALT (gut-associated lymphoid tissue) is to prevent the development of systemic infection by detecting and destroying infectious agents at an early stage [6]. It is believed that effectively functioning cellular mechanisms of the gut-associated lymphoid tissue (GALT) play a key role in birds’ resistance to coccidiosis. In addition, GALT contains B and T lymphocytes, which play a key role in birds’ acquired immunity to coccidiosis [6,7].

Coccidiosis is a parasitic disease caused by protozoa belonging to the subphylum Apicomplexa of the genus *Eimeria* found within the gastrointestinal tract [8]. It is mainly a disease affecting chickens, although there are also cases in other species, e.g., ducks or turkeys [9]. Several publicly available research results have shown that the incidence of infection increased among younger chicks, while older chickens were relatively resistant to infection [10,11]. Four-week-old chicks are more susceptible to caecal coccidiosis, while two-week-old chicks are more resistant than at any other time during the first six weeks, which may be due to differences in the natural immunity of the host or variability in the virulence of the inoculum used [11,12]. For most *Eimeria* species, they infect birds between 3 and 18 weeks of age, but relatively higher mortality rates have been reported in young chicks [11,13,14].

The genus *Eimeria* includes 1700 described species of protozoa, also found in invertebrates and vertebrates. These protozoa are widely distributed around the world [15]. Protozoa of the genus *Eimeria* are characterized by high specificity concerning the host species and the location in which they inhabit its organism. There are seven species of coccidia in chickens: *Eimeria tenella*, *Eimeria acervulina, Eimeria necatrix*, *Eimeria brunetii, Eimeria maxima, Eimeria mitis,* and *Eimeria praecox*, the last two of which are considered by many researchers to be ambiguously pathogenic species [16]. However, more and more data indicate that these species affect the profitability of production, because infection, despite the lack of visible symptoms of disease and deaths, leads to a deterioration in the economic results of breeding [8,17,18]. Each year, coccidiosis causes over 3 billion dollars in losses in global poultry production [19,20,21]. The global costs associated with this disease in the poultry sector can be as high as USD 2.4 billion per year [20]. In Poland alone, in 2005, losses related to the occurrence of coccidiosis amounted to about PLN 120 million [22]. By contrast, estimates in the European Union show that the total cost associated with coccidiosis for a broiler producer is around €0.05 per chicken, of which 70–80% is due to subclinical disease. This gives a loss of 2000 euros with a herd of 40,000 broilers [23]. Subclinical coccidiosis is one of the biggest challenges in intensive poultry farming, as indicated by numerous studies documenting the high occurrence of *Eimeria* species responsible for this disease, such as *Eimeria maxima* and *Eimeria acervulina* [15]. Due to the constantly increasing poultry production, especially in the sector of chickens for fattening, a further increase in the risk of coccidiosis is expected. It is estimated that the subclinical form of the disease is responsible for up to 70% of all costs associated with coccidiosis [24]. In addition, the lack of effective methods of rapid diagnosis of this form of the disease is a significant problem. In the literature, attempts can be found to search for links between the assessment of the degree of invasion using available diagnostic tools and production results, which could facilitate the detection of subclinical coccidiosis [25]. This problem is further exacerbated by the growing resistance to coccidiostats used. As early as 1951, Horton-Smith predicted the phenomenon of the development of resistance, and the following years brought numerous reports of the ineffectiveness of some preparations. Because of these factors, it can be concluded that coccidiosis not only remains a serious problem in poultry production but will pose an even greater challenge in the coming years [15].

Coccidia in poultry reproduce in the epithelium of the gastrointestinal tract, and individual species prefer specific sections of it. *Eimeria tenella* is located in the cecum, *Eimeria acervulina* inhabits the duodenum, *Eimeria brunetti* in the rectum and cecum, while *Eimeria maxima* and *Eimeria necatrix* live in the small intestines [8]. Coccidiosis can take a clinical form or a subclinical form [26]. Depending on the species of coccidia, the symptoms and strength of pathogenesis may vary slightly. The clinical form of the disease is characterized by weakness, lethargy, drowsiness, and spiky plumage. Birds often adopt a “vulture-like” posture. Initially, the feces are yellowish or brick in color, which then changes to a chocolate-mahogany color, containing fresh shreds of blood, which is characteristic of the *Eimeria tenella* invasion. In the case of infection with *Eimeria brunetti* and *Eimeria necatrix*, feces become brownish pink, foamy, liquid, or brown, with a paste-like consistency. Increased thirst is also observed in chickens. Anatomopathological changes during *Eimeria tenella* infection include distension of the cecum, hemorrhagic mucosal inflammation and the presence of a significant number of blood clots in their lumen. Pallor of skeletal muscles is also a characteristic feature. Invasion with the most pathogenic species of chicken coccidia, *Eimeria necatrix*, causes severe distension of the small intestine, which takes on a bluish red or red-raspberry color, and hemorrhagic-necrotizing inflammation of the intestinal mucosa. In the case of *Eimeria brunetti* infestation, fibrinous inflammation occurs in the large intestine, in the stalk and in the initial parts of the iliac and cecum sections. Characteristic clinical symptoms and anatomopathological changes facilitate the diagnosis of the disease. On the other hand, infestations caused by *Eimeria maxima* and *Eimeria acervulina* lead to a subclinical form of the disease, which is manifested by a decrease in body weight gain and an increase in the feed conversion ratio (FCR), which negatively affects the economic efficiency of rearing. In this form, however, it is more difficult to identify the direct effect of *Eimeria* spp. on breeding results, as other diseases can also contribute to the deterioration of production parameters. *Eimeria praecox* and *Eimeria mitis* are considered to be low-pathogenicity species, although new studies indicate an increased risk of subclinical disease when *Eimeria praecox* and *Eimeria mitis* are present together [15,27].

Microscopically, coccidia invasion is manifested by granulocyte and mononuclear cell infiltration, as well as swelling and thickening of the intestinal mucosa. Cell infiltration in chickens is dominated by CD8+ T cells, which often form large clusters in the lamina propria of the mucosa and intestinal crypts [28]. The course of the disease depends on the *Eimeria* species, its pathogenic capacity, the amount of invasive oocysts ingested by birds, the age of the birds, and the birds’ resistance to coccidiosis [16].

**Scheme 1:**
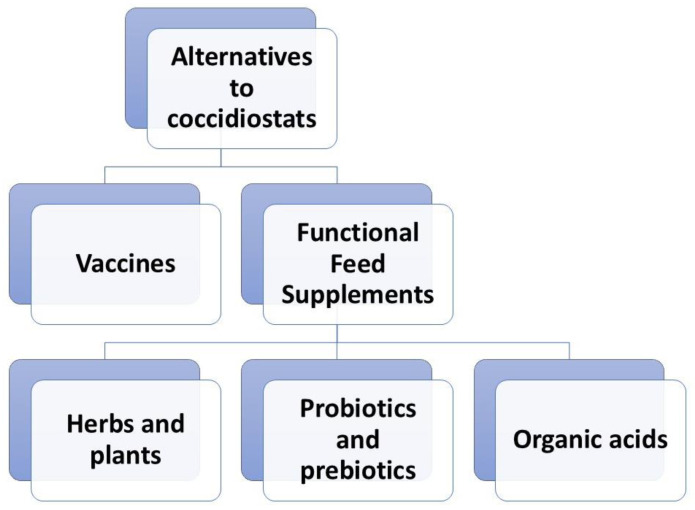
Alternatives to coccidiostats are being discussed in the review.

In broiler breeding, coccidiostats have so far been commonly used to prevent coccidiosis by inhibiting or killing coccidia [29], and some of them have additional antibacterial properties. In response to growing restrictions on the use of antibiotics in animal production, substitutes are being sought that, on the one hand, must protect the animal’s digestive tract from colonization by pathogenic microorganisms and, on the other hand, be neutral to its organism [30]. This function can be performed by vaccines and functional feed supplements discussed in the review of available literature (Scheme 1).

## 2. Coccidiosis

### 2.1. Life Cycle of Eimeria

**Scheme 2:**
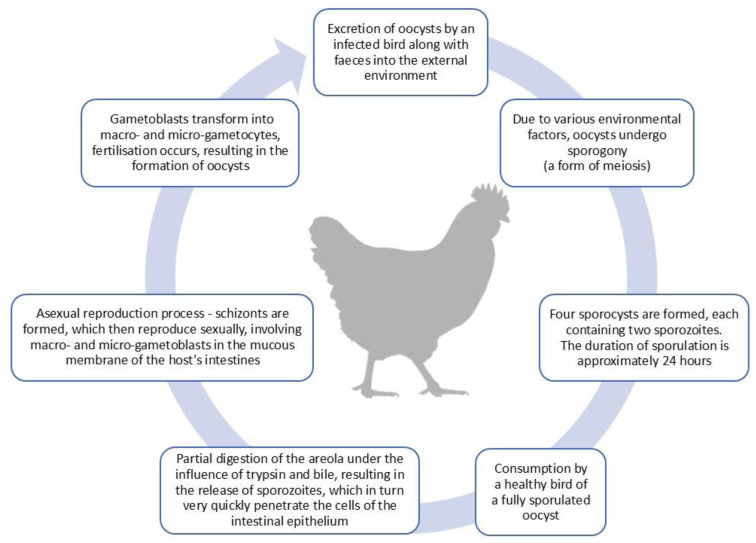
Simplified scheme of the life cycle of *Eimeria* [32,33].

The life cycle of coccidia was first described in 1910 by Harold Fantham [31]. In broiler chickens, the development cycle of protozoa of the genus *Eimeria* is monoxenic and is closely related to the host’s gastrointestinal tract. The invasive form is oocysts, which are round or oval in shape, and their dimensions range from 15.6 to 30.5 μm in length and from 14.2 to 20.7 μm in width, depending on the specific species [15]. The life cycle (Scheme 2) begins with the oocyst stage, which, together with the feces, is excreted into the external environment (litter or soil). Only under the influence of various environmental factors, such as the right temperature, the presence of oxygen, and humidity, oocysts undergo the process of sporogony, which is a form of meiosis. As a result, 4 sporocysts are formed in the oocyst, and each of them contains 2 sporozoites. The duration of the sporulation depends on the species and is usually around 24 h. Only an oocyst that has gone through the full process of sporulation can develop further. After the bird ingests the sporulated oocyst, the areolas surrounding it are partially digested by the action of trypsin and bile, which leads to the release of sporozoites. These, in turn, penetrate the cells of the intestinal epithelium very quickly [32]. As a result, the next stage occurs, which is the process of asexual reproduction. In this stage, schizonts are formed, which then reproduce sexually, with the participation of macro- and microgametoblasts within the intestinal mucous membranes of the host. Later, the gametoblasts are transformed into macro- and microgametocytes. Then fertilization occurs, as a result of which oocysts are formed, which, together with feces, end up in the external environment, thus closing the developmental cycle [33].

### 2.2. Prevention of Coccidiosis

Currently, chemoprophylaxis, using coccidiostatic preparations in feed, remains one of the main methods of preventing coccidiosis. This state of affairs has been maintained for over 60 years. Initial research on coccidiostats concerned not only their antibacterial properties, but also the antiparasitic properties of sulfonamides [34]. The results of these studies laid the foundation for further search for coccidiostats, which led to the discovery of new substances [15]. Chemoprophylaxis consists of administering coccidiostats to broilers throughout the breeding period, excluding the withdrawal period before slaughter. Coccidiostats available on the market are divided into two main groups: products of actinobacterial fermentation from *Sterptomyces* spp. and *Actinomadura* spp. (e.g., narasin, monensin, lazalocid, maduramycin, salinomycin and *semduramicin*) and chemical coccidiostats (e.g., nicarbazine, diclazuril, robenidine, halofuginone) [35,36]. To slow down the development of coccidia resistance to these preparations, a rotation program is used, in which the coccidiostat is changed every 2–3 cycles, or an exchange program in which different coccidiostats are used: one in the starter feed and the other with a different mechanism of action in the grower feed [37]. It is worth remembering that in the case of chemical coccidiostats, resistance to these substances develops quite quickly. For example, preparations such as quinolones, which include decoquinate, which is one of the chemical coccidiostats, are relatively safe, have no effect on egg production, egg quality or feed efficiency, but unfortunately, resistance to them builds up relatively quickly. Therefore, as part of rotational programs, they are usually used only once a year [15,38].

Alternative or complementary methods of combating coccidiosis include active immunoprophylaxis, which involves vaccinating broiler chickens, and passive immunoprophylaxis, which involves vaccinating the parent flock [39].

Another method of combating coccidiosis that is gaining importance is the addition of plant supplements or herbal preparations to feed or water. Also, when describing the methods of coccidiosis prevention, it is impossible to ignore the immunomodulatory effect of preparations that are prebiotics and probiotics, as well as the use of organic acids.

Over time, it has been observed that proper flock management (related to avoiding overcrowding, maintaining proper litter conditions, and ensuring adequate ventilation for the birds) has a key impact on the risk of coccidiosis-related problems [40].

Therefore, strict compliance with the principles of biosecurity is applied following the Regulation of the Minister of Agriculture and Rural Development of 18 September 2003 on detailed veterinary requirements to be met by farms where animals or animal products from these farms are placed on the market, concerning:

- isolating individual poultry houses by using separate staff, feeding, and tools;
- applying the principle “the whole poultry house full—the whole poultry house empty”;
- maintaining appropriate conditions in terms of temperature, access to light, humidity, and air exchange;
- protecting fodder against the access of wild birds and rodents;
- keeping a register of third parties entering the farm;
- using the right stocking in the poultry house;
- isolating poultry in one poultry house, keeping the herd at a uniform age;
- regular disinfestation, disinfection, and pest control, along with recording these activities;
- disinfection of the wheels of vehicles entering the farm;
- regular updating of the rodent protection plan [41].

## 3. Coccidiostats

### 3.1. Modes of Action

Coccidiostats are classified into two basic groups. The first group consists of natural compounds produced by bacteria of the *Streptomycetaceae* family, referred to as polyether ionophores. Their chemical structure includes tetrahydrofuran rings connected to spiroketal fragments. The second group consists of coccidiostats, often referred to as chemical coccidiostats, including compounds such as quinolones, pyridones, guanidines, thiamine analog and triazine derivatives and other chemicals [42].

**Scheme 3:**
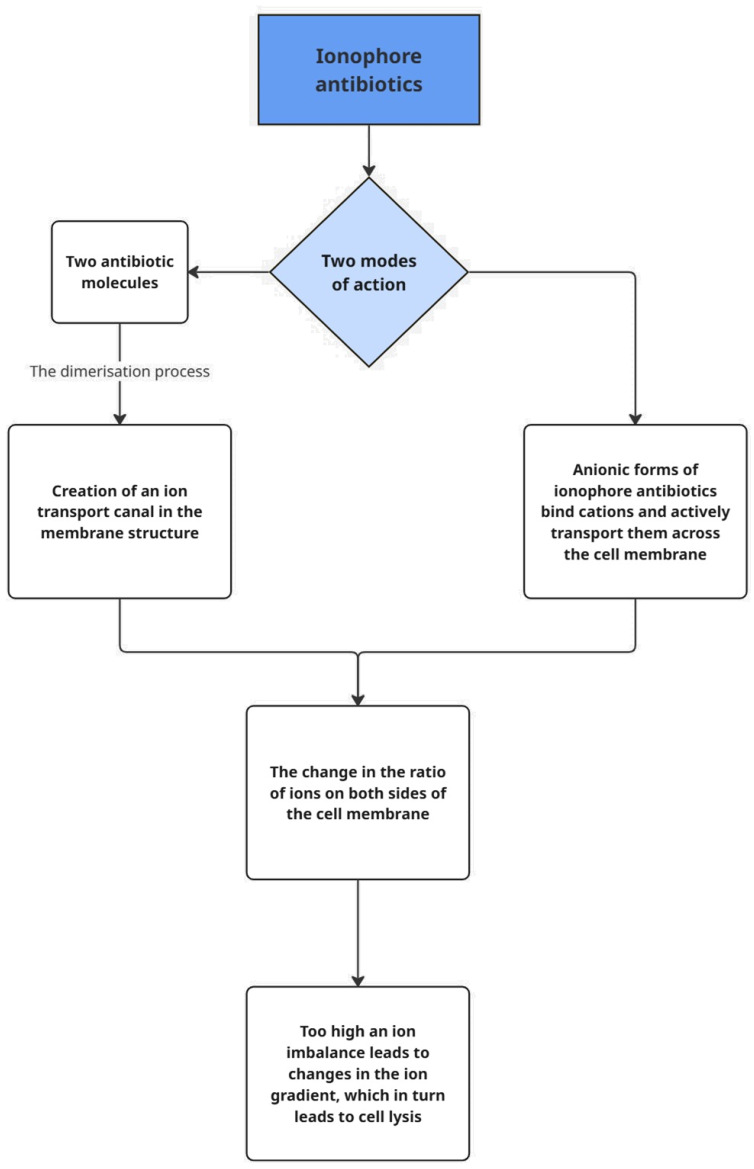
Simplified scheme of two modes of action of ionophore antibiotics [43,44].

Ionophore antibiotics have two mechanisms of action (MoA) (Scheme 3), which, despite their differences, lead to a common biological effect—an imbalance in ion concentrations on both sides of the cell membrane. In the first mechanism, antibiotic molecules form dimers that organize themselves into channels that allow cations to be transported through the lipid bilayer. The second mechanism involves anionic ionophores binding cations and actively transporting them across the cell membrane. After crossing the membrane, cations are released into the cytoplasm [43,44]. Under physiological conditions, cells have defense mechanisms against such disturbances. Enzymes such as Na^+^/K^+^-ATPase and Ca^2+^/Mg^2+^-ATPase regulate the appropriate concentrations of ions on both sides of the membrane. The action of ionophore antibiotics disrupts this balance [45].

Chemical coccidiostats work in different ways. The decant interferes with electron transport in the mitochondria of protozoa, which inhibits the development of the parasite. Robenidine and nicarbazine are thought to inhibit energy production in mitochondria, although their mechanism of action requires further investigation. Very little information is available in the reference literature on the mechanisms of action of halofuginone and diclazuril [44,46].

### 3.2. Toxic Effect

The threat posed by coccidiostats affects both humans and animals. Monitoring of the toxicity mechanisms of these substances in farm animals began at an early stage of their use, with particular emphasis on the effect of ionophores on nervous tissue. Numerous studies have shown that poisoning usually occurs after safe doses were exceeded, as exemplified by salinomycin poisoning in turkeys and rabbits or monensin poisoning in broilers. Accidental exposure can also lead to poisoning in other species, such as dogs and horses. The most commonly observed clinical symptoms include loss of appetite, diarrhea, impaired coordination, stiff gait and reluctance to move, muscle tremors, myoglobinuria, apathy and weakness. Poisoning with ionophores can be acute, leading to death within a few hours (e.g., within 7 h), or chronic, with symptoms of congestive circulatory failure [44]. Since the latter part of the 1990s, there has been growing interest in the presence of coccidiostats in food, as reflected in the increasing number of reports of food contamination with, among others, nicarbazin and lasalocid [42]. Coccidiostats appear in foods of animal origin due to non-compliance with withdrawal periods, contamination of supposedly drug-free feeds, human error, or imperfect manufacturing processes and handling procedures. It is also possible to transmit these substances between animals (through fecal ingestion). Acute poisoning in humans is rare, but possible. As a result of consuming food contaminated with coccidiostats, several people experienced symptoms such as vomiting and weakness in the lower limbs within four hours. Further clinical course included polyneuropathy, rhabdomyolysis, hypercalcaemia and respiratory failure, which led to death in two cases. However, the few incidents described confirm the effectiveness of existing safety procedures in preventing food contamination with coccidiostats and in maintaining their concentrations in food products at levels considered safe. Nevertheless, the growing scale of food production requires the development of more effective methods for detecting and controlling coccidiostat residues and the consistent implementation of these procedures in practice. Reports indicate that cooking contaminated food does little to reduce the concentration of these substances. Moreover, ionophores have a narrow safety range, which increases the risk of poisoning. The cases described indicate an important need for effective monitoring of food for the presence of coccidiostats or their residues [44].

### 3.3. Causes of Coccidiostats in Food

In most cases, the presence of coccidiostat residues in foods of animal origin is due to human error. They most often appear during the production of premixes and feed, as well as during the administration of drugs to animals. It happens that breeders deliberately use feed containing coccidiostats throughout the fattening period, fearing the negative impact of changing feed before slaughter on the condition of animals. Coccidiostats may also interact with other drugs, e.g., ionophore antibiotics with thiamulin. Such interactions slow down the metabolism of ionophores, which results in a longer period of persistence of residues in animal tissues [47]. Individual biological factors, such as the altered ability to absorb, metabolize, distribute, or excrete coccidiostats, are also important. Factors such as age, sex, differences in metabolism, or the presence of diseases can lead to the accumulation of these substances or their metabolites in the animal’s body [48]. Under certain breeding conditions, the phenomenon of secondary circulation of coccidiostats and their active metabolites (recycling) may also occur. This is particularly relevant in broilers raised on deep litter, where birds may ingest active substances previously excreted in the feces. Even if an appropriate grace period is maintained before slaughter, this phenomenon may lead to the occurrence of residues in tissues [47].

### 3.4. Resistance to Coccidiostats in Coccidia

Since 1939, when sulfanilamide was shown to control coccidiosis in chickens, the industry has increased the use of similar (chemical) compounds. To this group of compounds, sulfachinoxaline was quickly added, followed by nitrofurazone and 3-notroxarson, amprolium, and nicarbazin [49].

However, the development of resistance has become a key issue associated with the use of coccidiostats. The first communication on the possibility of resistance to anticoccidial drugs was presented by Horton-Smith in 1952 in an abstract presented at the 9th World Congress in Paris, France [50]. As presented by Joyner et al. [51], resistance was not a serious problem in the control of coccidiosis in the field. Currently (in the 21st century), the situation has changed significantly, with many drugs being introduced into circulation, to which resistance has developed. In a study on resistance to anticoccidial drugs, according to Abbas et al. [52], since the publication of the first study by Waletzky et al. [53], many anticoccidial drugs have been used and resistance to all of them has been reported in various parts of the world. However, it is suggested that resistance to ionophore coccidiostats develops at a slower rate than to non-ionophore coccidiostats. One proposed explanation for this slow acquisition of resistance to ionophores may be that they allow some leakage of susceptible oocysts, which in turn leads to less rigorous selection for resistance than in the case of non-ionophore coccidiostats. Previous opinions assumed that in order to minimize the occurrence of resistance, it is crucial to shorten the exposure time to anticoccidial drugs as much as possible. In addition, it was suggested to alternate between different coccidiostats with different mechanisms of action in successive flocks, combine non-ionophore coccidiostats with ionophore therapy, or use swing programs during flock rearing [32,54].

Coccidiostats can be added to different feeds in the form of different programs:

- single-drug, where the coccidiostat is a single drug administered throughout the life of the entire flock,
- shuttle, where several coccidiostats (ionophores or synthetic compounds) are used in different feeds within a single flock. However, the results of studies conducted on birds administered monensin alone or in shuttle programs with various synthetic drugs were inconclusive [50,55,56,57].

Currently, one of the main issues that continues to be discussed among those involved in coccidiosis is the ability of organisms to acquire resistance to one drug through the use of another drug, known as cross-resistance [58]. According to Chapman [50,59], ‘multiple resistance is resistance to more than one drug, even if they have different mechanisms of action.’ Therefore, it is important to note that cross-resistance and multiple resistance are not similar.

## 4. Alternative Methods: Vaccination and Functional Feed Supplements

### 4.1. Vaccine

One of the most commonly used alternative methods of combating coccidiosis in commercial poultry flocks is vaccination. This has become a response to the growing resistance to available coccidiostats and consumer concerns about the presence of these substances in meat or eggs [15].

The first attempts at immunoprophylaxis against coccidiosis were made at the beginning of the 20th century, and in 1952, the first commercial vaccine was introduced to the market in the USA. Since then, research into vaccines against coccidiosis has been ongoing.

The ideal coccidiosis vaccine for broilers should meet several requirements:

1. Efficacy in inducing immunity against economically important *Eimeria* species.
2. Safety for animals, other species and people.
3. No negative impact on the environment.
4. The content of low-virulence strains that displace more virulent strains.
5. Storage stability, even under sub-optimal conditions.
6. Protection against wild strains of coccidia from different geographical regions.
7. Accurate, effective and easy dosage capability.
8. No side effects.
9. Compatibility with other poultry vaccines.
10. No pollution (viruses, bacteria, fungi, chemical pollution).
11. Economic competitiveness compared to other methods of coccidiosis control.
12. Presence of drug-sensitive strains that may displace resistant strains [60].

The effectiveness of vaccines largely depends on the correctness of their administration [61]. Most coccidiosis vaccines are given to birds in drinking water. Studies have shown that birds that are infected with a small number of *Eimeria* spp. oocysts develop resistance to coccidiosis after two or three consecutive infections [62]. It has also been proven that repeated mild coccidiosis infections result in more effective stimulation of the immune system than a one-time, strong infection [63]. When administering a live vaccine, birds are vaccinated with live oocysts to provide an early initial stimulus for the immune response. After the birds are placed on litter, new vaccine oocysts are excreted in the feces and, after passing through the sporulation stage, can reinfect the flock. It is assumed that secondary exposure to vaccine oocysts and wild-type oocysts present in the litter can induce protective immunity. The development of immunity takes several weeks, and some cases of vaccination failure are due to birds being exposed to virulent wild-type oocysts before they have had time to develop an immune response. Of course, it is important that vaccination is carried out with extreme caution, as chicks that are not exposed to vaccine oocysts may be susceptible to potentially large numbers of virulent oocysts when placed on litter. The use of this form of vaccination is intended to induce sufficient immunity to prevent chronic infection, while allowing sufficient *Eimeria* to accumulate to develop a full immune response to local *Eimeria* species [20,64,65,66,67].

When choosing vaccines, it is important to remember that the immunity of birds to coccidiosis is species-specific. This means that there is no cross-immunity between different species of the parasite. Scientific work carried out on the processes involved in the immune response against *Eimeria* spp. has shown a wide variation in this response depending on the coccidia species and suggested that sporozoites of different species recognize differentiated host cell structures during the infection process [15,68,69]. In addition, studies on the course of the immunomodulatory response of the most economically important species have shown differences between them in, for example, the expression of cytokines, which play one of the most important roles during immune formation [15,70].

This has made it difficult to design a vaccine that would induce simultaneous immunity to all major *Eimeria* spp. This has made it difficult to design a vaccine that induces simultaneous immunity to all major *Eimeria* spp. For this reason, it is necessary to use a vaccine that protects against several *Eimeria* species with which the birds may come into contact during the production cycle.

Studies have proven that it is possible to simultaneously produce immunity to different species of coccidia, which allows the use of vaccines containing different species of *Eimeria* in their composition [71].

Active immunization of poultry against coccidiosis does not provide full protection against natural infection, but significantly reduces its intensity. This is manifested by a decrease in the number of oocysts excreted and a reduction in damage to epithelial cells in specific parts of the intestines [63]. This indicates a small role of specific antibodies in preventing the development of infection caused by coccidia. Humoral immunity seems to act mainly against the extracellular stages of the parasite’s development. The role of intestinal secretory antibodies is in reducing the adhesion of coccidia to the intestinal epithelium. Studies conducted by Lillehoj [72] indicate a small role of B lymphocytes and humoral response in coccidia infections, in which it was found that hormonal or chemical bursectomy of chickens does not change the course of primary and secondary infection with coccidia pathogenic to poultry.

Among the vaccines against coccidiosis, live vaccines can be distinguished:

(1) fully virulent (non-attenuated),
(2) fully virulent attenuated,
(3) resistant to ionophore coccidiostats,
(4) for in ovo administration and subunit vaccines with completely different principles of action than live vaccines [63].

Currently, the most commonly used coccidiosis vaccines on the European market are live attenuated vaccines containing isorulated oocysts of several species, which vary from vaccine to vaccine. In their formulation, these vaccines contain different doses of islandorulated oocysts, previously attenuated by repeated passage of the parasite on chicken embryos (for example, *E. tenella* in the Livacox^®^ vaccine) or by selection of “early maturing” strains, so-called “precocious lines” (the other species in the Livacox^®^ vaccine and all strains in the Paracox^®^ vaccine [15,73,74]. Due to the reduction in the number of schizogony generations, the “early maturing” strains are characterized by a shortening of the endogenous phase of the development cycle, which also has the consequence of reducing the number of oocysts excreted with feces while the birds gain immunity [15,74,75]. The Hipracox^®^ vaccine, which also contains “early maturing” strains, is also commercially available on the European market. It should be noted, however, that the first vaccines introduced against coccidiosis were live vaccines and were not subject to any modification to reduce their pathogenicity. Such vaccines include, for example, Immucox^®^, Nobilis^®^Cox ATM or Inovocox^®^. These vaccines are still available in many markets [15].

Assessing the effectiveness of coccidiosis vaccines is a major challenge in practice. The key importance is attributed to the colonization of litter with oocysts, which promotes an increase in the number of vaccine oocysts in the environment and, at the same time, reduces the occurrence of field strains [65]. According to the results presented by Williams and co-authors [76], in flocks of vaccinated birds, the number of oocysts in the litter peaked at 21 and 35 days of age, while in flocks receiving coccidiostats, the peak of oocyst counts was recorded only at 35 days of age, where it exceeded the level observed in vaccinated birds. Other studies have shown that the maximum number of oocysts in litter occurred only at 21 days of age [15].

### 4.2. Herbs and Plants

#### 4.2.1. Herbs and Phytogenic Feed Supplements in the Diet of Poultry

Herbal feed supplements are currently gaining popularity. Plant products that are natural, less toxic, and free of residues are considered to be ideal growth stimulators to include in the diet of animals [77]. Phytogenic feed supplements, one of these alternatives, are also attracting increasing interest due to their positive effects on improving growth rates, reducing susceptibility to stress, and enhancing the immune response.

Herbs and their extracts represent a relatively new category of supplements in poultry feed. Traditionally, they have been used primarily to enhance animal health and feed quality, but only recently have they been considered as agents for improving animal productivity. Their nutritional value lies in the presence of biologically active compounds that influence the body, enhance feed flavor, stimulate appetite, and affect gastrointestinal motility and enzyme secretion—factors that positively impact both metabolic and production processes [30,78]. The inclusion of herbs and plant-based supplements in animal diets has also been shown to strengthen immunity, reduce disease incidence, and promote animal welfare. Although herbs do not provide direct nutritional value, they enhance the palatability and digestibility of feed, leading to more efficient nutrient utilization. Advances in research have enabled the identification of active compounds in herbs and a better understanding of their effects, allowing for more targeted and rational use in animal nutrition.

Herbs contain a wide array of bioactive constituents, such as flavonoids, anthocyanins, glycosides, tannins, mucilages, bitters, essential oils, alkaloids, terpenes, triterpenes, saponins, iridoids, naphthoquinones, anthraquinones, phenols, phenolic acids, and mineral salts [30,79,80]. These compounds exhibit diverse effects, including immunostimulatory, anti-inflammatory, and antibacterial properties, and can also contribute to improving the quality of animal products. Herbs stimulate digestive juice secretion, increase appetite, improve intestinal motility, and enhance nutrient absorption [80]. However, unlike antibiotic growth promoters, herbs generally produce beneficial effects more gradually. Increasingly, plant-derived compounds—known as phytobiotics—are being used in animal nutrition to support productivity and improve product quality. These substances are valued for being natural, safe, and versatile, offering a broad range of applications in animal husbandry [81].

Herbs contain a variety of biologically active substances that exhibit a wide spectrum of activity. Administered as supplements to feed in the form of a mixture, they complement each other, which allows for a stronger effect than when using single substances isolated from plants. In breeding chickens in natural conditions, herbs such as dandelion, yarrow, nettle, mint, sage, goose cinquefoil, knotweed, lemon balm, St. John’s wort and many others are added to the feed. Poultry eat them willingly and tolerate them well. These plants are characterized by anti-inflammatory, astringent, disinfectant and immunity-supporting properties. Due to the presence of bioactive substances, such as polyphenols (mainly responsible for antioxidant activity), chlorophyll, essential oil components, ascorbic acid, or carotenoids (to a lesser extent), these herbs improve the palatability of fodder and support their digestion [80].

#### 4.2.2. Effect of Herbs and Plant Feed Supplements in Feed on the Welfare and Conditions of Poultry Farming

Many studies confirm the positive effects of herbs on poultry health. Herbs, by enhancing taste and stimulating appetite, act as regulators of digestive functions (e.g., thyme, cumin). They have a protective effect (e.g., flax), support metabolism (e.g., fenugreek, knotweed), and also have antidiarrheal, antibacterial, and anti-inflammatory (e.g., garlic, onion, sage), antifungal (e.g., lavender), and anabolic (e.g., onion, garlic) properties. Herbs reduce susceptibility to stress, strengthen the immune system (e.g., echinacea), and neutralize the negative effects of antinutrients [82]. Biologically active substances present in herbs, such as essential oils, tannins, glycosides, flavonoids, terpenes, mucilage, or organic acids, are characterized by antibacterial, antiviral, antifungal, immunostimulatory, and anti-stress properties. In addition, they improve the taste and aroma of animal products, give an intense color to egg yolks, and bind mycotoxins [80].

Herbs can be introduced into feed in various forms, such as:

- single herbs or herbal mixtures, both fresh and dried,
- licks, infusions, decoctions, decoctions essential oils and mixtures of natural volatile compounds,
- herbal preparations containing a variety of plant substances in the form of aqueous, alcoholic or hydroalcoholic extracts, often standardized for the content of specific active ingredients [83].

Studies indicate that multi-component herbal preparations are an effective alternative to antibiotic growth promoters in poultry nutrition. In vitro and in vivo studies have confirmed the antibacterial activity of plants such as *Origanum vulgare*, black pepper *(Piper nigrum*), clove (*Syzygium aromaticum*) and thyme (*Thymus vulgaris*), as well as the components of essential oils (e.g., thymol, carvacrol, curcumin, piperine, eugenol) against various pathogenic bacteria, such as *Clostridium perfringens*, *Escherichia coli*, *Staphylococcus aureus, Salmonella* Typhimurium, *Listeria monocytogenes* or *Yersinia enterocolitica* [30,80].

#### 4.2.3. Plants, Herbs and Substances Contained in Them as Agents with Coccidiostatic Properties

The use of plant extracts in the treatment of coccidiosis is not a new approach. When choosing the right plant extract to fight this disease, it must be taken into account that it must be at least partially lipid-soluble to penetrate cell membranes, since coccidia develop inside cells. Two Chinese plants, *Dichroa febrifuga* and *Sophora flavescens*, which are rich in alkaloids, are effective in treating coccidiosis [84].

In addition, a mixture of extracts of sage (*Salvia officinalis*), garlic (*Allium sativum*), purple coneflower (*Echinacea purpurea*), thyme (*Thymus vulgaris*) and oregano (*Origanum vulgare)* has a positive effect on the production results of birds infected with *Eimeria* spp. oocysts [80].

Thymol and carvacrol are the most important bioactive compounds contained in thyme. Their percentage content in thyme oil is 28.53% and 25.06%, respectively [85,86]. In addition to the aforementioned thymol and carvacrol, thyme is also a rich source of linalool, terpineol, borneol, cineol, and bornyl acetate. These substances reduce the number of harmful intestinal microorganisms and have antiseptic and antioxidant properties. Furthermore, they reduce the risk of diarrhea and have a beneficial effect on weight gain and feed conversion [30,86]. The phenolic bioactive substances contained in thyme also increase the secretion of endogenous digestive enzymes (proteases, amylases, lipases) and stimulate poultry immunity [86,87]. These compounds also exhibit strong anticoccidial properties against *Eimeria tenella, Eimeria acervulina,* and *Eimeria maxima*. Thymol and carvacrol, which belong to the phenol group, alter the permeability of the cytoplasmic membrane to cations (H+ and K+), thereby impairing important cellular processes. The resulting leakage of cellular components leads to an imbalance in the cell’s water content, a collapse of the membrane potential, inhibition of ATP synthesis, and ultimately cell death. Due to their toxic effect on the upper layer of mature enterocytes of the intestinal mucosa, they accelerate the natural renewal process, resulting in the shedding of sporozoite-infected cells before the coccidia reach the merozoite stage [11].

Saponins contained in many plants, including *Acacia concinna*, disrupt the lipid structure of the parasite’s cell membrane by binding to membrane cholesterol. The direct impact on enzymatic activity and metabolism leads to cell death, which in turn causes a toxic effect in mature enterocytes in the intestinal mucosa. As a result, cells infected with sporozoites are released before the protozoa reach the merozoite stage. In addition, saponins strengthen non-specific immunity and increase production efficiency (through increased daily weight gain and better FCR, lower mortality). They contribute to reducing oocyst excretion in feces and reducing ammonia production [11].

*Yucca schidigera* extract, which is a source of natural saponins, can also be used to prevent coccidiosis. These saponins inhibit the growth of protozoa by interacting with cholesterol in the cell membrane of parasites, leading to their death. In addition, yucca extract supports the efficacy of vaccination against coccidiosis, controlling protozoan invasion and stimulating the development of immunity [88].

In addition, saponins (found, e.g., in *Glycyrrhiza glabra*, *Gypsophila paniculate*, *Aesculus hippocastanum*) are used to coat Eimeria antigens in immunostimulatory complexes during vaccine preparation [11].

Another group of biologically active compounds found in plants are alkaloids. They have shown significant potential as natural antibiotics with a broad spectrum of antibacterial activity, minimal side effects and a reduced risk of drug resistance [89,90]. For example, sanguinarine, which belongs to the alkaloid group, induces apoptosis in *E. tenella* sporozoites through reactive oxygen species, reduction in mitochondrial membrane potential and an increase in calcium ion concentration [90,91].

Berberine, which also belongs to the broad group of alkaloids, shows anticoccidial activity, which has been demonstrated in diets supplemented with berberine in broilers infected separately with different species of Eimeria (*E. acervulina*, *E. maxima*, *E. tenella*, *E. mitis* and *E. praecox*). Based on the results obtained, it was concluded that this bioactive compound reduces the excretion of oocysts in feces in most *Eimeria* species, although it was less effective in the case of *E. maxima* [90,92].

Phenolic compounds such as curcumin or diferuloylmethane contained in *Curcuma longa* (turmeric) have an inhibitory effect on the growth of selected *Eimeria* species (*Eimeria tenella*, *Eimeria acervulina*, and *Eimeria maxima*) in the sporogonic phase (destroying sporozoites), thus preventing sporozoite invasion into intestinal epithelial cells and the merogonic phase. This reduces oocyst excretion and intestinal damage [11].

Proanthocyanidins from grape seed extract can inhibit the development of coccidiosis due to their anti-inflammatory properties, reducing the severity of lesions in the cecum. This, in turn, reduces mortality and improves production results. However, the effect depends on the dose—optimal results were observed at a dose of 10 mg/kg of extract, while higher concentrations increased mortality [93].

The addition of green tea extract also has a positive effect. Green tea, rich in polyphenols called catechins, has anticancer, anti-inflammatory, antioxidant, antibacterial, antiviral, and antiparasitic properties. It was proven that chickens fed with 0.5% and 2% green tea powder excreted 50% less oocysts in the feces [94].

### 4.3. Probiotics and Prebiotics

One possible way to prevent coccidiosis is to use preparations containing probiotics and prebiotics. In animal production, probiotics have been defined as feed supplements consisting of live microorganisms that benefit the animal’s body by enhancing the microbial balance in the gut, leading to benefits such as improved feed conversion rate, nutrient absorption, growth rate and economic aspects of poultry production [95,96]. Prebiotics are defined as dietary components that are not digested in the digestive tract of monogastric animals. They stimulate the growth or activity of beneficial intestinal microbiota, thereby positively influencing the health status of the host organism [97,98].

In this prevention, bacteria of the genera *Enterococcus*, *Pediococcus* and *Lactobacillus* are most often used, as well as probiotic products containing *Bifidobacterium* and *Pediococcus*. According to the study, birds infected with different species of *Eimeria* spp. that received probiotics containing *Enterococcus* bacteria showed greater weight gain compared to birds that were not given probiotics. In addition, these birds had a milder course of diarrhea after infection, and this group had the lowest mortality [99]. *Pediococcus*-based probiotics are the most effective in protecting against *Eimeria acervulina*, which is responsible for small bowel coccidiosis, although they have not been effective against *Eimeria tenella*, which causes cecum diseases [100]. These results highlight the selective effects of probiotics in different parts of the poultry digestive tract. *Lactobacillus*, which is the most abundant group of lactobacilli, are among the most commonly used microorganisms in the production of probiotics. Research by Dallul and colleagues [101] showed that probiotic preparations containing *Lactobacillus* increase the number of intraepithelial lymphocytes in the intestines. On the other hand, research conducted by Ritz et al. [102] proves that the administration of probiotics alone can reduce changes in the intestines caused by various species *of Eimeria* spp. However, the best results in preventing coccidia infections can be achieved by using probiotics and vaccines together.

Unlike probiotics, prebiotics support the growth of beneficial bacteria already present in the digestive tract. Most often, prebiotics are produced based on mannan oligosaccharides derived from the cell walls of the yeast *Saccharomyces cerevisiae* [15]. These substances support the increase in the height and integrity of intestinal villi, as well as modulate intestinal and systemic immunity [103]. A particularly important aspect in the prevention of coccidiosis is the ability of mannan oligosaccharides to stimulate the production of bacteria of the genera *Bifidobacterium* and *Lactobacillus*, which create unfavorable conditions for the development of coccidia and *Salmonella* [104].

In addition, the use of probiotics and prebiotics in broiler chicken nutrition also brings a number of benefits related not only to reducing the risk of coccidiosis, but also to improving health, production performance, and the quality of animal products.

The use of the probiotic strain *Bacillus amyloliquefaciens* (BAP) in direct feeding at a rate of 20 g/kg of feed mixture during 35 days of rearing had a positive effect on the growth performance of broiler chickens. This may have been due to the good health of the birds’ intestines, which improved the digestion and absorption of nutrients provided in the feed [105]. The introduction of probiotic supplements in the form of pure strains, including *Bifidobacterium animalis*, *Enterococcus faecium*, *Bacillus subtilis animalis*, *Lactobacillus reuteri animalis* or mixtures consisting of several probiotic strains at a dose of 5 × 108 CFU/kg of feed mixture improved the growth rates of broiler chickens [96,106]. The use of the *Bacillus subtilis* strain (GalliPro^®^) at a dose of 0.2 g/kg of feed reduced the requirement for amino acids and crude protein, and consequently the cost of feed for broilers [96,107]. In addition, supplementation with probiotic microorganisms has been effectively used to reduce the content of pathogenic bacteria that appear during meat loading and in the subsequent processing and packaging of meat products [96,108].

The positive effects of prebiotics on the broiler chicken organism include improving appetite and the efficiency of animal production, improving gastrointestinal motility and secretion of digestive juices, and reducing the risk of diarrhea in animals. They also stimulate the immune system, which may indirectly reduce mortality in young animals. Prebiotics regulate the pH of the digestive tract, have a protective, antiseptic, and antioxidant effect, regulate metabolism, and improve the quality of animal products. These supplements stimulate intestinal mucus secretion and limit the population of bacteria from groups such as *Escherichia*, *Salmonella*, *Streptococcus*, *Staphylococcus*, *Pseudomonas* and *Clostridium* [97,109,110].

The beneficial effects of prebiotics on broiler chicken health are related to their ability to:

- inhibiting the colonization of the bird’s intestine by pathogenic bacteria,
- adsorbing microorganisms and their toxins on its surface,
- prolonging the passage time of food contents,
- delaying gastric emptying—‘feeling of satiety’,
- stimulating the secretion of intestinal peptide hormones,
- reducing glucose absorption,
- lowering the pH of the gastrointestinal tract,
- regulating fecal production (frequency and consistency) [109,111].

### 4.4. Organic Acids

Organic acids are a promising alternative to ionophore antibiotics for the prevention of coccidiosis. The use of supplemental organic acids as dietary supplements and water acidifiers resulted in an increase in the number of jejunal goblet cells, which leads to stimulation and production of a mucus layer [112,113]. The goblet cells are the most important structure responsible for the production of mucins, which play the role of the first line of defense by maintaining the intestinal barrier.

Abbas et al. [114] demonstrated the effect of acetic acid on *E. tenella* in broiler chickens. Acetic acid lowers the pH of the cecum and reduces the impact of oocysts, ultimately reducing inflammatory bowel changes.

The use of Activate^®^ DA, containing calcium 2-hydroxy-4-butyrate (Ca(HMTBa)_2_), fumaric and benzoic acid at a dose of 2%, or Lacplus at a dose of 1%, which contains lactic, citric, fumaric and phosphoric acids, in chickens, may be an alternative method of controlling coccidiosis in broiler flocks. Studies have shown that their use leads to a decrease in intestinal pH, reduced oocyst excretion and increased weight gain in birds [115,116].

The addition of a mixture based on microencapsulated organic acids, such as formic, phosphoric, lactic, acetic, butyric and propionic acids, with the addition of glycerol and silicon dioxide, can act as an effective coccidiostatic to feed. According to the study, the use of such a mixture in broilers significantly reduces the amount of oocysts excreted on the 42nd day of rearing compared to the control group without supplementation [116,117].

According to the results of a study published by Ali et al. [114], the inclusion of butyric acid glycerides in feed reduced the number of inflammatory intestinal lesions caused by *Eimeria* coccidia. In addition, in the case of coccidiosis infection, the administration of a probiotic (Primalac) in combination with butyric acid glyceride resulted in a reduction in the number of oocysts excreted compared to birds that did not receive this combination. This suggests that such a combination of ingredients may have a coccidiostatic effect [116,118].

## 5. Conclusions

According to available data, global meat production is expected to continue increasing in line with steady population growth, with the poultry sector projected to experience particularly dynamic expansion.

Poultry meat consumption is rising significantly, driven by its favorable nutritional properties, relatively low cost, and comparatively reduced environmental footprint. At the same time, consumer awareness of healthy eating and the demand for food products with high nutritional and health value are growing. Along with the increase in consumption, there is also growing social pressure to reduce the use of chemotherapeutic agents in broiler raising, which has also extended to coccidiostats. Consequently, there is an urgent need for safe and effective alternatives that protect consumer health while supporting poultry production in line with both industry requirements and consumer expectations.

**Scheme 4:**
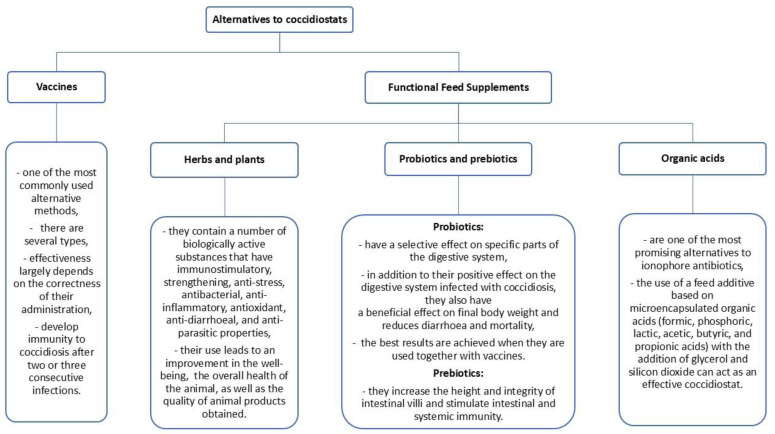
Summary of the review article in the form of a simplified scheme (based on the content of the manuscript).

Observing the efforts of the scientific community in the search for a “golden mean,” it can be concluded that progress is steadily being made toward this goal. The growing body of literature provides increasingly extensive data on the safety and effectiveness of vaccination strategies for controlling coccidiosis in broiler chickens. At the same time, functional feed supplements—such as plants and herbs and their active compounds, prebiotics, probiotics, and organic acids—are gaining prominence (Scheme 4). These alternatives are not only promising tools for coccidiosis prevention but may also contribute to reducing the global problem of antibiotic resistance.

This raises the question of whether vaccines and functional feed supplements together can be considered a “golden mean” that meets both consumer and producer expectations while limiting the incidence of coccidiosis. With ongoing scientific advancements, this question is likely to be answered in the near future. However, continued research is essential to fully elucidate the mechanisms of action, additional benefits, and potential risks associated with alternatives to coccidiostats in broiler chicken raising.

## Data Availability

No data was used for the research described in the article.

## Abbreviations

The following abbreviations are used in this manuscript:

ATP	Adenosine Triphosphate
FCR	Feed Conversion Ratio
EC	European Commission
EU	European Union
EFSA	European Food Safety Authority
GALT	Gut-Associated Lymphoid Tissue
MoA	Modes of Action
MRLs	Maximum residue Limits
PLN	Polski Zloty
USD	United States Dollar
